# The clinical effectiveness of sivelestat in treating sepsis patients with both acute respiratory distress syndrome and septic cardiomyopathy

**DOI:** 10.1186/s13019-024-02835-3

**Published:** 2024-06-27

**Authors:** Hui Lv, Langjing Huang, Xiuhong Yang, Changdong Zhang, Hao Yu, Xiaoke Shang

**Affiliations:** 1 Department of ICU, Wuhan No.1 Hospital, No.215 Zhongshan Avenue, Wuhan, China; 2 Department of Cardiovascular Medicine, Changsha Economic Development Zone Hospital, Changsha, China; 3 Department of Cardiovascular Surgery, Union Hospital, Tongji Medical College, Huazhong University of Science and Technology, No.1277 Jiefang Avenue, Jianghan District, Wuhan, 430022 Hubei Province China; 4 Department of Cardiology, Wuhan No.1 Hospital, No.215 Zhongshan Avenue, Wuhan, China

**Keywords:** Acute respiratory distress syndrome, Septic cardiomyopathy, Sivelestat, Echocardiography, Electrocardiogram

## Abstract

**Background:**

We aimed to assess the efficacy of the neutrophil elastase inhibitor, sivelestat, in the treatment of sepsis-induced acute respiratory distress syndrome (ARDS) and septic cardiomyopathy (SCM).

**Methods:**

Between January 2019 and December 2021, we conducted a randomized trial on patients who had been diagnosed with sepsis-induced acute respiratory distress syndrome (ARDS) and septic cardiomyopathy (SCM) at Wuhan Union Hospital. The patients were divided into two groups by random envelop method, the Sivelestat group and the Control group. We measured the serum concentrations of Interleukin (IL)-6, IL-8, Tumor necrosis factor-α (TNF-α), and High-mobility group box 1 (HMGB1) at five time points, which were the baseline, 12 h, 24 h, 48 h, and 72 h after admission to the ICU. We evaluated the cardiac function by sonography and the heart rate variability (HRV) with 24-hour Holter recording between the time of admission to the intensive care unit (ICU) and 72 h after Sivelestat treatment.

**Results:**

From January 2019 to December 2021, a total of 70 patients were included in this study. The levels of IL-6, IL-8, and TNF-α were significantly lower in the Sivelestat group at different time points (12 h, 24 h, 48 h, and 72 h). HMGB1 levels were significantly lower at 72 h after Sivelestat treatment (19.46 ± 2.63pg/mL vs. 21.20 ± 2.03pg/mL, *P* = 0.003). The stroke volume (SV), tricuspid annular plane systolic excursion (TAPSE), early to late diastolic transmitral flow velocity (E/A), early (e’) and late (a’) diastoles were significantly low in the Control group compared with the Sivelestat group. Tei index was high in the Control group compared with the Sivelestat group (0.60 ± 0.08 vs. 0.56 ± 0.07, *P* = 0.029). The result of HRV showed significant differences in standard deviation of normal-to-normal intervals (SDNN), low frequency (LF), and LF/HF (high frequency) between the two groups.

**Conclusions:**

Sivelestat can significantly reduce the levels of serum inflammatory factors, improve cardiac function, and reduce heart rate variability in patients with Sepsis-induced ARDS and SCM.

## Introduction

Sepsis is a severe medical condition that can lead to high rates of morbidity and mortality [1]. As sepsis becomes more severe, the likelihood of complications such as acute respiratory distress syndrome (ARDS)/ acute lung injury (ALI) and septic cardiomyopathy (SCM) increases [2, 3]. ARDS/ALI has been linked to sepsis for almost a century [4], with more than half of Intensive Care Unit (ICU) admissions for sepsis resulting in ARDS development [5]. SCM is a life-threatening condition that results in myocardial dysfunction and multiple organ failure, occurring in 40–60% of septic shock patients [6–8]. Despite significant progress in ARDS/ALI treatment [9], treatment options for SCM remain limited, resulting in a high mortality rate [10].

Sivelestat, a selective neutrophil elastase inhibitor, has shown promise in treating various conditions, including ALI, refractory Kawasaki disease, and acute pancreatitis [11]. Its protease-inhibiting and anti-inflammatory effects have been demonstrated as effective in shortening ICU duration and reducing ventilator use in ARDS/ALI patients [12]. Additionally, studies suggest that Sivelestat can reduce and prevent tissue ischemia and reperfusion injury (IRI) in multiple organs [13]. Earlier studies in Japan have confirmed that the effects of sivelestat in reducing the permeability of pulmonary blood vessels [14], inhibiting mucus secretion in the epithelial layer [15], and decreasing the production of inflammatory cytokines such as IL-1β, IL-6, and TNF-α at clinically available concentrations [16] and protecting against postperfusion-induced lung injury. Although the specific mechanisms of Sivelestat’s role in sepsis are not yet fully understood, previous studies have pointed to its anti-inflammatory, anti-oxidative stress, and anti-apoptotic properties [17]. The present study aims to determine the therapeutic effect of Sivelestat in treating sepsis-induced ALI and SCM, with the goal of improving patient outcomes and reducing mortality rates.

## Materials and methods

### Design, patients, and grouping

A prospective randomized controlled study was conducted on patients with Sepsis-induced ARDS/ALI and SCM, who were admitted to the ICU from January 2019 to December 2021. Patients under the age of 18, those with severe liver cirrhosis, previous liver transplantation, severe central nervous system disease, uncontrolled malignancy, or clotting disorders were excluded from the study [18]. The Sivelestat group and the Control group were classified based on whether patients received the Sivelestat administration or not and it was determined by the random envelop method. This study was approved by the ethics committee of Wuhan Union Hospital (No. 2018-0598-08) and was registered on the China Clinical Trial Registry (ChiCTR-OPC-17,013,126). It was performed in accordance with the 1964 declaration of Helsinki and later amendments. All methods were carried out in accordance with relevant guidelines and regulations. Informed consent was obtained from all subjects and/or their legal guardians.

### Diagnosis of sepsis-induced ARDS/ALI and SCM

According to the criteria established by the Third International Consensus Definitions for Sepsis and Septic Shock (Sepsis3), we defined sepsis as a condition where an infection triggers a life-threatening host response leading to organ dysfunction [19, 20]. We measured organ dysfunction by monitoring changes in Sequential Organ Failure Assessment (SOFA) scores that an increase in score of 2 points or more was considered indicative of dysfunction [21]. To diagnose ARDS, we followed specific criteria: 1) severe hypoxemia (PaO_2_/FiO_2_ ≤ 300 mmHg), 2) acute symptoms that developed within a week, 3) bilateral radiographic abnormalities (not attributable to atelectasis), and 4) The disorder was not caused by heart failure [22]. Meanwhile, we diagnosed SCM based on three criteria as follows: First, the patient had been diagnosed with sepsis; Second, the patient had one of the following three ultrasound abnormalities: A) left ventricular systolic mitral annulus velocity (LV-Sm) < 8 cm/s or Left Ventricular Ejection Fractions (LVEF) < 50%; B) Right ventricular systolic mitral annulus velocity (RV-Sm) < 12 cm/s; or C) peak early diastolic transmitral flow velocity/peak early diastolic mitral annular velocity (E/e’) > 15 or peak early diastolic mitral annular velocity (e’) < 8 cm/s; Third, the patient had no history of chronic heart disease, including coronary heart disease, chronic heart failure, regional ventricular wall motion abnormality, dilated cardiomyopathy, hypertrophic obstructive cardiomyopathy, congenital heart disease, or heart valve disease [23].

### Interventions and treatment

Each ICU physician made antibiotic selections that were appropriate for the patients and a low dose of steroids was given to manage septic shock. Patients diagnosed with ARDS and SCM received intravenous injections of Sivelestat (Shanghai Huilun Pharmaceutical Co., LTD, China) at a rate of 0.2 mg/kg/hr on the day of diagnosis [24] and the treatment was continued until either the patient was discharged from the ICU or a period of 14 days. Accordingly, patients in the Control group were under the same treatment regiments such as cardiac function and respiratory function maintenance, which were consistent with those in the Sivelestat group, except that patients did not receive the Sivelestat.

### Outcomes

The Clinical Effectiveness of Sivelestat was evaluated based on plasma inflammatory factor levels, cardiac function, and heart rate variability (HRV), among which the cardiac function evaluated by sonography was the primary outcome. The patients’ test results indicating abnormal liver and kidney function were considered as adverse events.

At baseline and 4 additional time points (12 h, 24 h, 48 h, and 72 h after Sivelestat administration), clinical data were collected. The data included infection sites, assessment system values (such as the SOFA score, Acute Physiology and Chronic Health Evaluation II [APACHE II] score, and Systemic Inflammatory Response Syndrome [SIRS] score), and previous medical history. Blood samples were drawn, centrifuged, and stored at -80 °C [25]. Lab data, including levels of Serum Tumor Necrosis Factor-alpha (TNF-α), Interleukin-8 (IL-8), Interleukin-6 (IL-6), and High Mobility Group Box 1 (HMGB1), were measured at different time points via enzyme-linked immunosorbent assay (ELISA) by Wuhan ADANTI Biotechnology Co., Ltd.

### Cardiac function evaluated by sonography

At the time of ICU admission and 72 h after the Sivelestat treatment, a GE system equipped with a multiplane 5-MHz transesophageal echocardiographic transducer was used for Cardiac Function Evaluated. LV systolic function was evaluated using visual gestalt method, and Simpson’s method, fractional shortening. The visual gestalt method qualitatively assessed LV function based on LV size, contractility, and thickening of myocardial segments, and was classified into hyperdynamic, normal, mild, moderate, and severe LV dysfunction. Using Simpson’s method, readings were taken in apical 4-chamber view to calculate Left Ventricular End Diastolic Volume (LVEDV), Left Ventricular End Systolic Volume (LVESV), and Left Ventricular Ejection Fraction (LVEF) [26]. Tissue Doppler Imaging was used to assess mitral annular flow velocity, and lateral mitral annular flow velocities, and annular velocities during early (e’) and late (a’) diastoles were evaluated. Early to late diastolic transmitral flow velocity (E/A) was calculated to assess diastolic function, and E to early diastolic mitral annular tissue velocity (E/e’) was calculated to estimate LV filling pressures [27]. Additionally, in patients with Regional Wall Motion Abnormalities (RWMA), both lateral and septal mitral leaflet annular velocities were measured and averaged. Tricuspid Annular Plane Systolic Excursion (TAPSE) was used to evaluate right ventricular systolic function. Moreover, the Tei index was calculated by the pulsed Doppler method as (a-b)/b. These ultrasound measurements were conducted by an experienced ultrasound specialist to avoid inter-observer bias, and to avoid intra-observer bias, an average of three consecutive recordings were taken.

To eliminate any inter-/intra- observer bias, an average of three consecutive recordings were taken and the average results were measured by an independent attending sonographer. The results and readings would be reviewed by another independent attending sonographer, and if there was a significant discrepancy, the head of the Ultrasound Department would make a final decision.

### Electrocardiogram assessment

At the time of admission to the ICU (Baseline) and 72 h after the administration of Sivelestat, all patients underwent a 24-hour Holter recording using 3-channel real-time tape recorders. HRV was evaluated in both time and frequency domains [28]. During the analysis, only normal heartbeats were recorded, and all artifacts were removed. In the time domain, HRV parameters including the standard deviation of all R-R intervals (SDNN), the mean of the standard deviations of all R-R intervals for all 5-minute segments (SDANN), the square root of the mean squared differences of successive R-R intervals (RMSSD), and the percentage of adjacent normal-to-normal intervals differing by over 50 milliseconds (PNN50) were calculated [29]. In the frequency domain, heart rate power in the low-frequency range (0.04–0.15 Hz) (LF) and in the high-frequency range (0.15–0.40 Hz) (HF) were estimated. Additionally, the ratio of LF/HF was also calculated [30].

To eliminate any inter-/intra- observer bias, an average of three consecutive recordings were taken and the results were calculated automatically. Two independent researchers verify the final calculations and ultimately take the average of the three measurements.

### Sample size calculation and statistical analysis

With the cardiac function index as the primary outcome, the 15.0 version of PASS software was used to calculate the sample size according to the pre-trial results. When there were 35 people in each group, the sample size could meet the statistical power of α = 0.05, β = 0.8.

All statistical analyses were performed using SPSS 24.0. Continuous variables are presented as mean ± standard deviation (SD). Analysis of serum levels, which included HMGB1, IL-6, IL-8, and TNF-a, was conducted using repeated measures analysis of variance (ANOVA) to compare within-/between- group differences. Nonparametric continuous variables were analyzed by the Mann-Whitney U-test. Categorical variables were examined using the χ^2^ test. Statistical significance was defined as *P* values < 0.05.

## Results

**Fig. 1:**
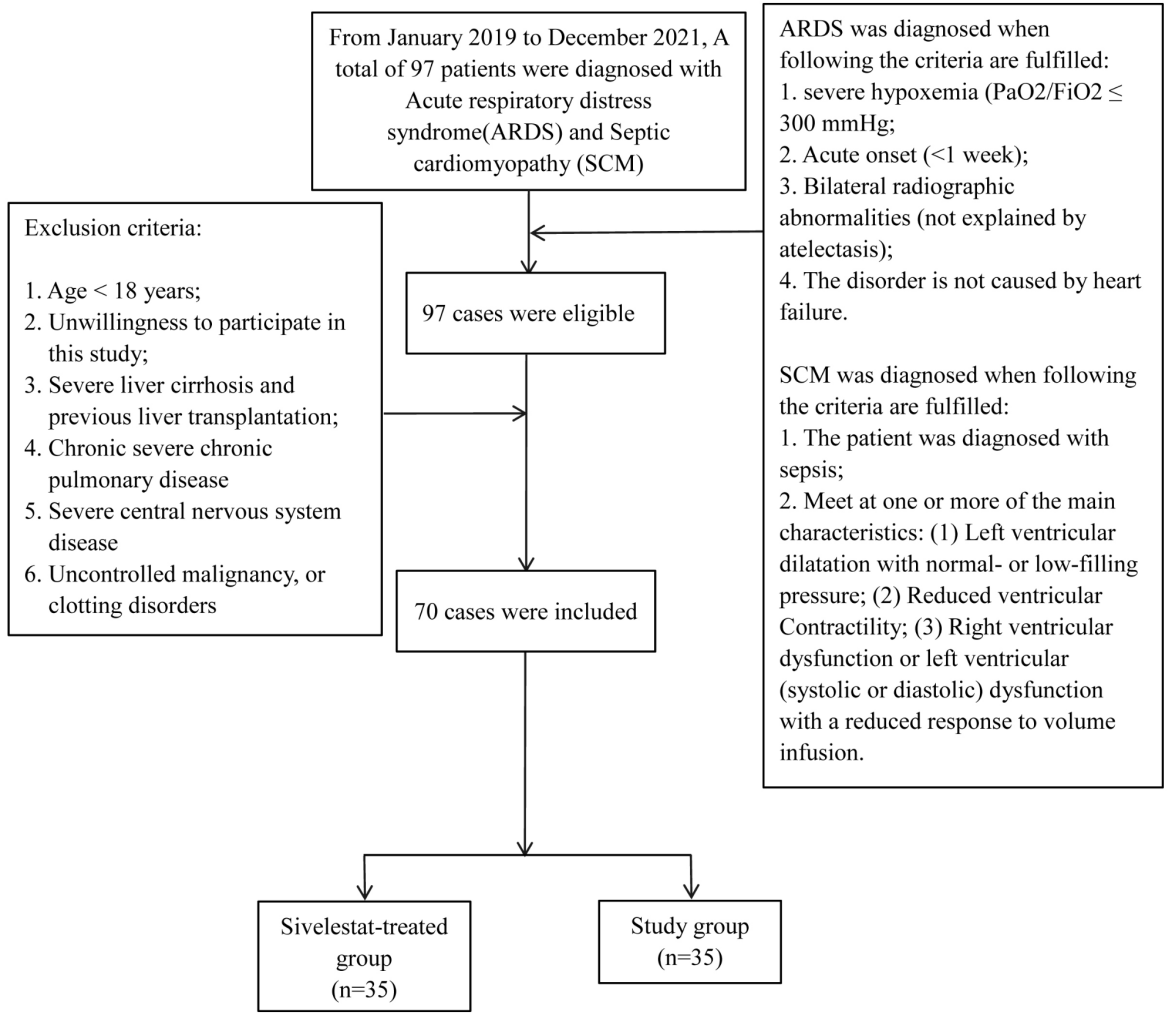
The enrollment flow chart

From January 2019 to December 2021, A total of 97 patients were diagnosed with acute respiratory distress syndrome (ARDS) and septic cardiomyopathy (SCM). According to the inclusion and exclusion criteria, 70 people were eventually included with 35 in each group. (Fig. 1.)

### Demography

No differences in age, body mass index (BMI), APACHE II score, SIRS score, SOFA score, and infection sites were observed between the two groups, *P* > 0.05. (Table 1).

**Table 1:** Characteristics of patients on baseline

	Sivelestat-treated group (*n* = 35)	Control group (*n* = 35)	χ ^2^ Value	*P* Value
**Age (year)**	58.54 ± 12.67	56.74 ± 14.83	0.849	0.399
**Gender, male/female**	19/16	21/14	0.233	0.629
**BMI (kg/m** ^**2**^ **)**	21.87 ± 5.46	20.75 ± 7.05	0.743	0.460
**APACHE II score**	24.52 ± 7.43	23.76 ± 6.39	0.459	0.648
**SIRS score**	3.25 ± 1.01	3.19 ± 1.26	0.220	0.827
**SOFA score**	13.37 ± 3.23	12.25 ± 3.07	1.487	0.142
**History, n (%)**				
Acute Heart failure	2 (5.71)	3 (8.57)	0.215	0.643
Cerebrovascular disease	1 (2.86)	3 (8.57)	1.061	0.303
Chronic lung disease	4 (11.43)	2 (5.71)	0.729	0.393
Chronic kidney disease	0 (0.00)	1 (2.86)	1.014	0.313
Hypertension	3 (8.57)	2 (5.71)	0.215	0.643
**Source of sepsis, n (%)**				
Pneumonia	6 (17.14)	8 (22.86)	0.357	0.550
Urinary tract Infection	14 (40.00)	12 (34.29)	0.245	0.621
Septicemia	4 (11.43)	6 (17.14)	0.467	0.495
Others	11 (31.43)	9 (25.71)	0.280	0.597

BMI: Body Mass Index; APACHE II: Acute Physiology And Chronic Health Evaluation II; SIRS: Systemic inflammatory response syndrome; SOFA: Sequential Organ Failure Assessment

### ELISA results

**Fig. 2:**
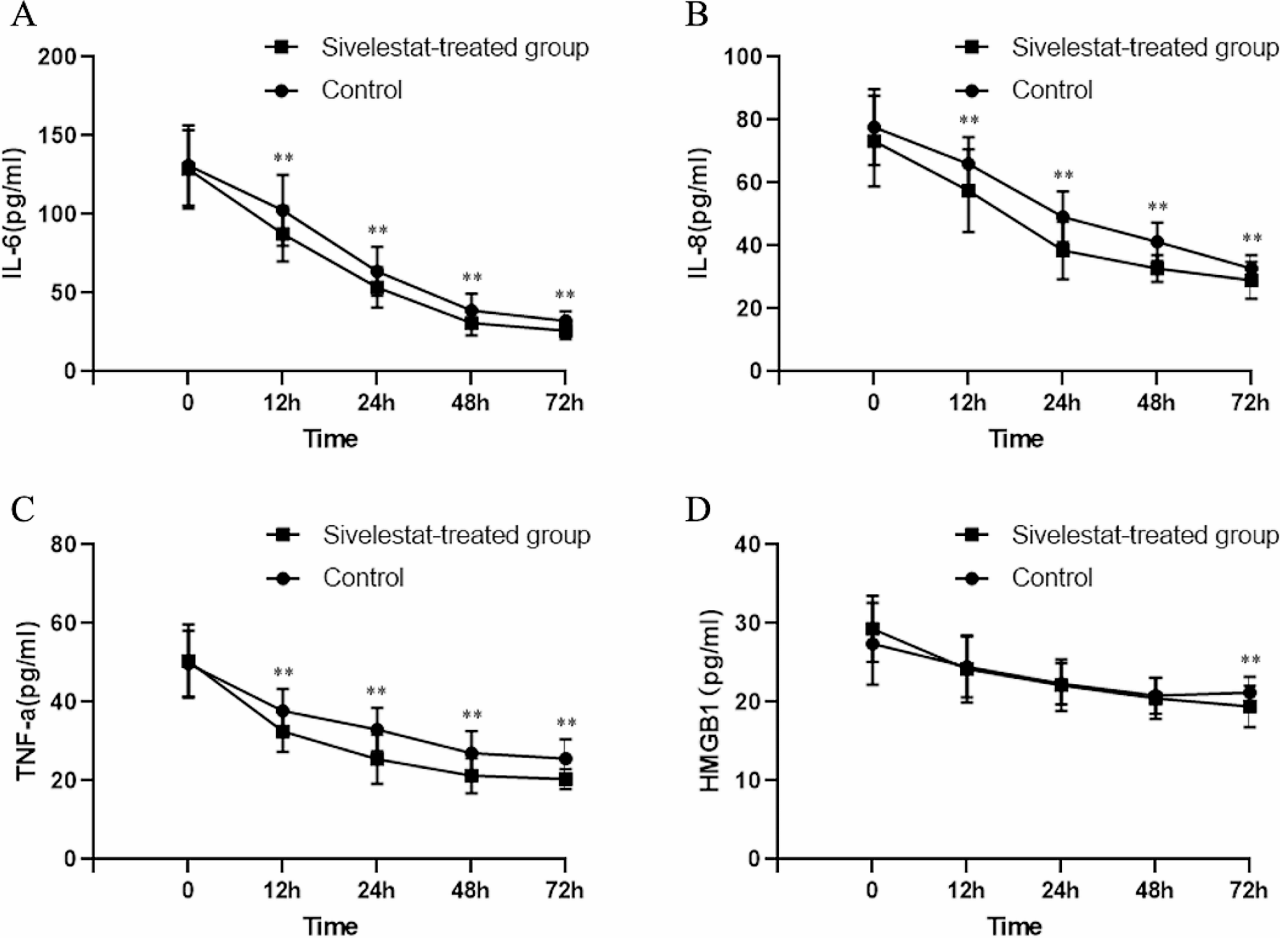
Changes of IL-6 (**A**), IL-8 (**B**), TNF-a (**C**), and HMGB-1 (**D**) before and after the treatment of Sivelestat IL-6: Interleukin-6; IL-8: Interleukin-8; TNF-a: Tumor necrosis factor-a; HMGB1: High-mobility group box 1 0 is the time of admission to the ICU (Baseline); 12 h, 24 h, 48 h, and 72 h are respective hours after taking the Sivelestat treatment. Data are presented as Mean ± Standard Deviation

The Sivelestat group exhibited significantly lower levels of IL-6 at various time points (12 h, 24 h, 48 h, and 72 h) compared to the Control group. The maximum difference was observed at 72 h after ICU admission (26.00 ± 5.39 pg/mL vs. 32.26 ± 6.13 pg/mL, *P* < 0.001; Fig. 2-A).

Similarly, IL-8 levels were significantly lower in the Sivelestat group at different time points (12 h, 24 h, 48 h, and 72 h), and the Sivelestat group exhibited significantly lower IL-8 levels at 48 h after ICU admission (32.83 ± 4.27 pg/mL vs. 41.26 ± 6.13 pg/mL, *P* < 0.001; Fig. 2-B).

The changes in TNF-a levels also showed a consistent trend with that of IL-6 and IL-8, and the levels were significantly lower at 72 h after ICU admission (20.40 ± 2.57 pg/mL vs. 25.60 ± 4.94 pg/mL, *P* < 0.001; Fig. 2-C).

No significant difference was observed in HMGB1 levels between the two groups at baseline, 12 h, 24 h, and 48 h. However, the Sivelestat group showed significantly lower levels of HMGB1 at 72 h after ICU admission (19.46 ± 2.63pg/mL vs. 21.20 ± 2.03pg/mL, *P* = 0.003; Fig. 2-D).

### Evaluation of cardiac function

**Fig. 3:**
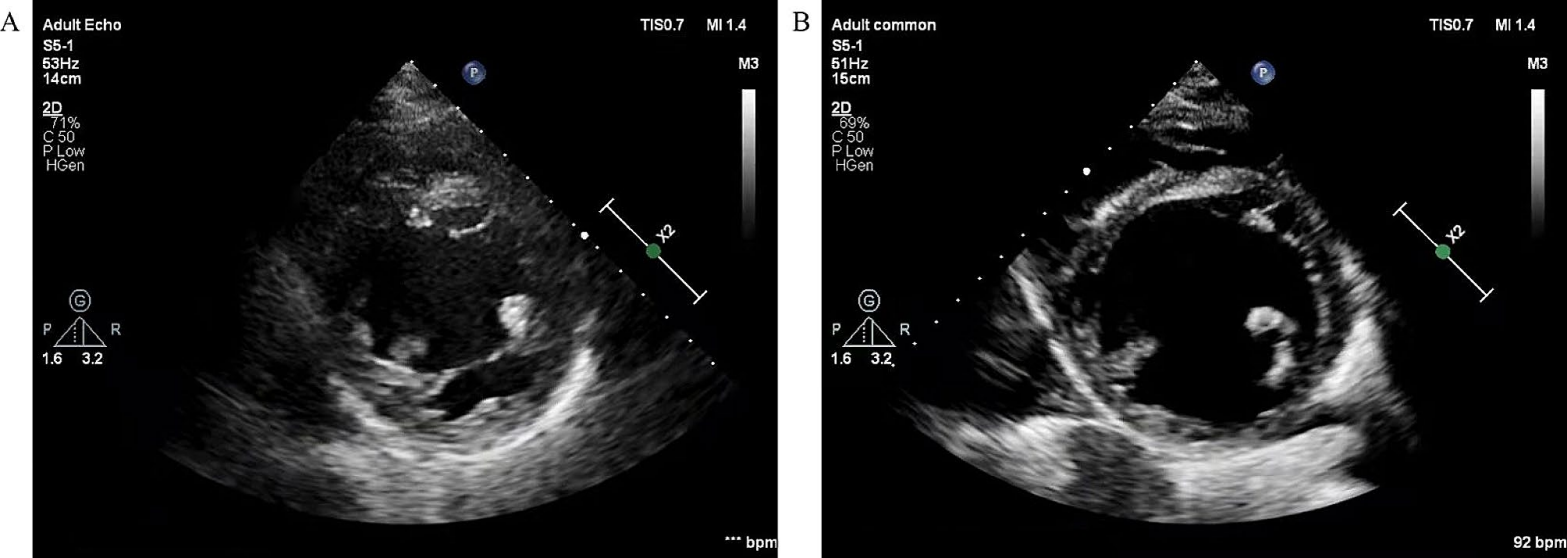
Echocardiographic imaging from Parasternal left ventricular short axis view. (**A**) Echocardiographic imaging at ICU admission. (**B**) Echocardiographic imaging at 72 h after the Sivelestat treatment

The echocardiographic features of two groups have been presented in Table 2. The comparisons of reduced peak early diastolic flow velocity (peak E), maximum inferior vena cava diameter (IVCmax), and minimum inferior vena cava diameter (IVCmin) showed no significant difference between the groups. However, the stroke volume (SV) was observed to be significantly lower in the Control group in comparison to the Sivelestat group (71.62 ± 12.32 ml vs. 78.68 ± 15.24ml, *P* = 0.037). Furthermore, the Tei index was significantly higher in the Control group in comparison to the Sivelestat group (0.60 ± 0.08 vs. 0.56 ± 0.07, *P* = 0.029), while the TAPSE value was significantly lower in the Control group in comparison to the Sivelestat group (16.63 ± 3.27 vs. 18.32 ± 3.62, *P* = 0.044). The Control group also exhibited significantly lower E/A, e’, and a’ values in comparison to the Sivelestat group, *P* < 0.05. The echocardiographic images in Fig. 3 demonstrate the efficacy of Sivelestat in treating SMC.

**Table 2:** Echocardiographic assessment between groups

	Sivelestat group (*n* = 35)	Control group (*n* = 35)	χ ^2^ Value	*P* Value
**E(m/sec)**	0.70 ± 0.13	0.75 ± 0.15	-1.490	0.141
**A(m/sec)**	0.87 ± 0.11	1.04 ± 0.09	-7.076	< 0.001*
**E/A**	0.88 ± 0.24	0.78 ± 0.17	2.012	0.048*
**e’(m/sec)**	0.55 ± 0.12	0.46 ± 0.15	2.772	0.007*
**a’(m/sec)**	0.76 ± 0.14	0.67 ± 0.09	3.199	0.002*
**E/e’**	9.63 ± 2.45	7.49 ± 2.12	3.908	< 0.001*
**ESV (ml)**	51.20 ± 11.42	60.34 ± 16.27	-2.720	0.008*
**SV (ml)**	78.68 ± 15.24	71.62 ± 12.32	2.131	0.037*
**EDV (ml)**	118.67 ± 37.21	138.59 ± 42.33	-2.091	0.040*
**TAPSE (mm)**	18.32 ± 3.62	16.63 ± 3.27	2.050	0.044*
**IVC max (mm)**	19.74 ± 4.31	21.45 ± 4.93	-1.545	0.127
**IVC min (mm)**	9.85 ± 2.56	10.47 ± 3.75	-0.808	0.422
**Tei Index**	0.56 ± 0.07	0.60 ± 0.08	-2.226	0.029*

ESA: end-systolic volume; SV: stroke volume; EDV: end-diastolic volume; TAPSE: Tricuspid Annular Plane Systolic Excursion; IVC: Inferior vena cava; Tei index: Myocardial performance index

*: Compared with the Control group, *P* < 0.05

### Comparisons about the electrocardiogram

The HRV indexes of the Sivelestat group and the Control group were presented in Table 3. There were no significant differences observed between the two groups, except for SDNN, LF, and LF/HF. The Control group exhibited a significant decrease in these three indices when compared to the Sivelestat group, *P* < 0.05.

**Table 3:** Comparisons of heart rate variability between the two groups

	SDNN (ms)	SDANN (ms)	rMSSD (ms)	PNN50(%)	LF (ms)	HF (ms)	LF/HF
**Sivelestat group (n = 35)**	115.45 ± 15.84	97.51 ± 10.27	34.57 ± 5.83	15.48 ± 4.57	8.58 ± 2.15	15.72 ± 5.15	0.53 ± 0.10
**Control group (n = 35)**	106.51 ± 12.72	98.26 ± 9.46	34.78 ± 8.48	14.84 ± 3.95	7.54 ± 2.07	15.27 ± 6.42	0.48 ± 0.08
**t Value**	2.603	-0.318	-0.121	0.627	2.061	0.323	2.310
***P *** **Value**	0.011*	0.752	0.904	0.533	0.043*	0.747	0.024*

SDNN: standard deviation of normal -to -normal intervals; SDANN: standard deviation of averaged normal -to -normal intervals; rMSSD: root of the mean square of successive differences; PNN50: percentage of adjacent normal-to -normal intervals differing by more than 50 ms; LF: low frequency; HF: high frequency

*: Compared with the Control group, *P* < 0.05

## Discussion

Sepsis is a condition where the body’s immune system responds abnormally to microbial infection resulting in systemic and dysregulated inflammation and the condition is associated with high mortality rates and can progress to sepsis syndrome and septic shock, which can be potentially lethal [31, 32]. The pathophysiology of sepsis and septic shock involves the cardiovascular system, although the underlying mechanisms of sepsis-associated myocardial depression remain unclear [33]. Recent research has elucidated several mediators that participate in the development of sepsis, including TNF-α, interleukins (IL), platelet activating factor (PAF), leukotrienes, thromboxane A2, and activators of the complement cascade [34, 35]. Toll-like receptor 4 (TLR 4) has also been implicated in myocardial depression caused by sepsis, particularly in recognizing bacterial endotoxin E, Gram-negative bacterial lipopolysaccharide (LPS), or lipooligosaccharide [36]. Myocardial depressant factors recognized to date include cytokines, the complement system, nitric oxide (NO), lipopolysaccharides (LPS), and high mobility protein box 1 (HMGB1), which include tumor necrosis TNF-α, interleukin-1β (IL-1β), interleukin-6 (IL-6), and HMGB1.

According to reports, Sivelestat, a specific inhibitor of neutrophil elastase (NE), has been found to not only inhibit NE itself but also cytokine production by monocytes [37]. Additionally, it has been found to inhibit nuclear factor kappa B (NF-κB), thereby decreasing the release of cytokines including HMGB1, regulating the production of IL-8 and MCP-1 in AEC-II [38, 39]. It appears that Sivelestat is capable of binding endotoxin or affecting the action of endotoxin-binding protein, thereby blocking endotoxin-triggered TNF-alpha production by macrophages both in vitro and in vivo, and inhibiting the binding of endotoxin to Toll-like receptors [40]. Sivelestat has also shown a protective effect on ischemia-reperfusion injury by inhibiting nitric oxide release during the ischemia and reperfusion phase [41]. This effect of Sivelestat resulted in the inhibition of nitric oxide-mediated vasodilation and the subsequent suppression of increased organ blood flow during reperfusion [42]. Moreover, Sivelestat has been found to inhibit the production of cytokines in culture systems even in the absence of neutrophils. Numerous studies have highlighted the beneficial effects of Sivelestat treatment in attenuating inflammatory and edematous responses in the lungs, mitigating acute lung injuries caused by endotoxins, mitigating mesenteric ischemia-reperfusion injuries, and reducing myocardial injuries following cardioplegic arrest [43]. Results of animal-based experiments have revealed that Sivelestat appears to have a cardioprotective effect against myocardial depression seen following global ischemia. The mechanism underlying the outcome could be associated with decreased formation of reactive oxygen species (ROS) and conservation of nitric oxide [44]. In another study, Toyama et al. reported an improvement not only in respiratory index but also the fractional area of the left ventricle during pediatric cardiovascular surgery with cardiopulmonary bypass, after administering Sivelestat [45]. Nonetheless, its effectiveness in preserving cardiac function during sepsis remains enigmatic in clinical practice. Although the promise of sivelestat is exciting, a number of questions remain. The timing and duration of sivelestat intervention may be crucial to its ultimate success. However, in the STRIVE study, some adverse events occurred, such as hypersensitivity, hepatobiliary disorders, blood and lymphatic system disorders, renal and urinary disorders. Although prespecified stopping guidelines were not met, a negative trend in long-term mortality prompted the DSMB to recommend suspension of enrollment and discontinuation of study drug [14]. To date, available clinical study data, including for the STRIVE study and the related postmarketing study, indicate no particular concerns regarding adverse events. The occurrence of adverse events must be confirmed in larger prospective RCTS and should be assessed over a longer period of follow-up.

Our study has demonstrated that administering Sivelestat immediately after SCM diagnosis resulted in a significant reduction in inflammatory factor levels such as IL-6, IL-8 and TNF-a, thereby improving SCM condition. These findings suggest that early Sivelestat treatment could be effective in managing SCM. Since the severity of organ dysfunction in SCM is associated with outcomes, attenuating SCM with Sivelestat may lead to a significant improvement in morbidity and mortality. Therefore, in our future studies, we plan to investigate the effect of Sivelestat on long-term prognosis and make a comparison to evaluate its effectiveness.

There are still some limitations in our study. First, statistical data related to prognosis are absent and no comparisons of mortality rate are made. Second, comparisons of treatment costs between groups are not studied. Third, the follow-up period is relatively short. In addition, the sample size of this study is relatively small due to the research topic, research funding and time constraints. We will increase the multi-center study and expand the sample size in the follow-up study to eliminate systematic bias and make the research results more rigorous and accurate.

## Conclusions

Sivelestat can significantly reduce the level of plasma inflammatory factors, improve cardiac function, and reduce heart rate variability in patients with sepsis-induced ARDS and SCM.

## Data Availability

No datasets were generated or analysed during the current study.
